# Sex differences in the early life stages of the salmon louse *Lepeophtheirus salmonis* (Copepoda: Caligidae)

**DOI:** 10.1371/journal.pone.0266022

**Published:** 2022-03-31

**Authors:** Andreas Borchel, Anna Zofia Komisarczuk, Frank Nilsen

**Affiliations:** Department of Biological Sciences, SLRC—Sea Lice Research Centre, University of Bergen, Bergen, Norway; Northumbria University, UNITED KINGDOM

## Abstract

Salmon lice are ectoparasites on salmonids and feed on blood, mucus, and skin from their hosts. This causes high annual costs for treatment and control for the aquaculture industry. Salmon lice have a life cycle consisting of eight life stages. Sex determination by eye is only possible from the sixth stage onwards. A molecular sex determination has not been carried out so far, even though few individual sex-linked SNPs have been reported. In the present study, we used known sex-specific SNPs as a basis to sequence the complete sex-specific gene variants and used the sequence information to develop a sex determination assay. This assay could be used to determine the developmental speed of the two sexes already in the earliest life stages. Additionally, we sampled salmon lice in the nauplius II stage, determined the sex of each individual, pooled their RNA according to their sex, and used RNA sequencing to search for differences in gene expression and further sex-specific SNPs. We succeeded in developing a sex-determination assay that works on DNA or RNA from even the earliest larval stages of the salmon louse after hatching. At these early developmental stages, male salmon lice develop slightly quicker than females. We detected several previously unknown, sex-specific SNPs in our RNA-data seq, but only very few genes showed a differential expression between the sexes. Potential connections between SNPs, gene expression, and development are discussed.

## Introduction

Salmon lice (*Lepeophtheirus salmonis* Krøyer, 1837) are parasites of Atlantic salmon and other Salmonids. They feed on their host’s blood, mucus, and skin, impairing the host’s health and growth [1]. Salmon lice are a significant problem in the aquaculture sector, causing high costs due to treatment and control to the industry every year [2].

The life cycle of salmon lice consists of eight different developmental stages separated by molts [3, 4]. After hatching from their egg, the first stage is the nauplius I stage, followed by the nauplius II and the copepodid stage. In these early larval stages, the lice are free-living and lecithotrophic. The copepodid is the infective stage and is the first stage to identify and attach to a host. On the host, the copepodids develop into chalimus I, chalimus II, preadult I and II, and finally, the adult stage. In the first description of the life cycle, four chalimus stages were identified [4]; however, a later study could prove that there are only two [3].

A morphological sex dimorphism becomes apparent to the naked eye in the preadult I stage [4] and becomes more pronounced within later stages. Females are larger than males, and the genital segment of both sexes is easily distinguishable by its shape. With an exact measurement of the cephalothorax length, one can also distinguish the sex of lice in the chalimus II stage. Already in this stage, females have a longer cephalothorax than males [5].

At the molecular level, differences between sexes are present at the mRNA [6, 7] and the microRNA [8] expression level. Especially genes involved in reproduction are expressed in a sex-biased way [9, 10]. Gene expression differences can be tracked back into the chalimus II stage and, in the case of one gene of unknown function, even into the copepodid stage [11].

At the genomic level, a sex-specific SNP in the prohibitin-2 gene is known [12]. In males, only guanine is found at the SNP’s position, while in females, guanine and thymine are present [12]. Additionally, females show a higher expression of the gene than males. More recently, more than ten sex-specific SNPs were identified in pacific salmon lice [13].

An additional distinction between the sexes is the developmental rate. It has been demonstrated that every molt after infestation occurs quicker for male than female salmon lice [14].

Furthermore, it has been shown that male and female lice react differently to some anti-louse treatments. Male salmon lice generally tolerate a higher concentration of emamectin benzoate than females [15]. Hence, sex should be considered (in all developmental stages) in various types of salmon lice studies to obtain accurate results.

The overall aim of the present study was to determine if there are sex differences present already in the earliest, free-living life stages of *L*. *salmonis*. In order to achieve this goal, we developed molecular markers to determine sex, as it is not possible to separate sex by eye at the early stages. Additionally, we investigated if the differences in developmental speed between sexes already occur as early as in the nauplius II stage. We also analyzed gene expression differences between female and male salmon lice at these early stages to identify candidate genes potentially involved in sex differentiation.

## Material and methods

### Animal culture & sampling

All salmon lice used were bred in the laboratory and belonged to well-established lab strains (LsGulen, LsOslo, Ls1A, described in [16]). As described in detail before [16], egg strings and planktonic life stages were kept in incubators in a flow-through system. The seawater used in these experiments is collected from the Byfjorden, outside of Nordnes, Bergen, Norway, from 105 m depth. The water has a salinity of roughly 34 ppt, and a temperature of 9°C, is filtered and UV-treated. The animals were kept on a 12h:12h light-dark cycle without any additions to the water. Lice used for RNA or DNA extraction were pipetted from their incubators into a Petri dish. As much water as possible was removed by pipetting. Lice used for RNA isolation were then submerged in RNAlater, kept in a fridge at 4°C for one day, and then frozen at -20°C, whereas lice used for DNA extraction were submerged in 70% ethanol.

Salmon lice as invertebrates are not under the animal experiment legislation. However, for their production Atlantic salmon were used as a host. This has been approved by the Norwegian Animal Research Authority (ID 8589).

### Identification of sex-specific variants

First, we examined the sequences of two genes with known sex-specific SNPs: prohibitin-2 (*phb2*) [12] and kinase suppressor of Ras 2 (*ksr2*) [13]. We amplified the region containing the known SNPs using PCR on cNDA obtained from female salmon lice. We sequenced these PCR products and observed additional heterozygous positions within the genes. PCR products were cloned into a TOPO-Cloning vector and introduced into TOP10-*E*. *coli*-cells. Sequencing of PCR products obtained from individual bacterial colonies allowed us to determine the sequences of individual variants. Thereby we could deduce variant-specific primers for 5’- and 3’- RACE (Smarter Race kit, Takara). After assembly of the sequences, we designed primers targeting the longest possible CDS-spanning sex-specific region. These primers were used for PCRs with the high-fidelity Q5-polymerase (NEB) and PCR-products sequenced. We also performed PCRs on genomic DNA to amplify the complete region containing the target gene. Sequencing was performed on an Applied Biosystem 3730XL Analyzer at the Sequencing facility at the University of Bergen. Genomic sequences and cDNA sequences were aligned to each other using Splign [17].

To verify the sex specificity of the different variants, we isolated genomic DNA from adult males and females of three different salmon louse strains using the GenElute Mammalian Genomic DNA Miniprep Kit (Sigma), following the manufacturer’s instructions. Again, Q5 polymerase was used to run a PCR for two variants of both genes in all DNA samples. A non-template- control was included. PCR products were visualized in 1% agarose gels, using GelRed and a gel documentation system.

In order to determine potential differences in gene copy numbers between males and females, qPCR was employed. 6.67 ng of genomic DNA extracted from male and female salmon lice of different breeding strains were used in assays specifically targeting each variant of *ksr2* and *phb2*. For each sample, the obtained gene copy numbers were then normalized by the respective gene copy numbers of Elongation factor *Ef1a*. The gene is likely autosomal, as it contains no known sex-specific SNPs. Thus, equal amounts of genomic DNA from females and males should contain an equal number of copies of this gene, allowing for correcting potential inaccuracies in the input amounts by normalization.

### Sex determination of larvae

We developed an assay for sex determination of the early larval stages from the salmon louse based on genomic DNA. To isolate DNA, we employed a slightly modified version of the HotSHOT DNA extraction method [18]. Nauplii and copepodids were harvested and stored in 70% ethanol. Individuals from one group (e.g., one egg string) were stored together in one reaction tube.

Single individuals immersed in storage solution (total volume 3 μl) were individually pipetted into PCR tubes containing 10 μl of lysis buffer (25 mM NaOH, 0.2 mM EDTA, pH 12). The PCR tubes were incubated for 1 hour at 95°C. After cooling down, samples were neutralized by adding 10 μl of Neutralizing solution (40 mM Tris-HCl, pH 5). Final samples containing the isolated genomic DNA were stored at 4°C or frozen at -20°C until further use. For the PCR assay, we used GoTaq G2 DNA polymerase (Promega). Each reaction contained 2 μl reaction buffer, 0.8 μl MgCl, 0.2 μl of the four primers 5’-TGTGCTAAAGTCAAATCAAGTTCG-3’, 5’-TGATTAGATTGTGGTGATATTCGGTA-3’, 5’-TGGTGATATTTTGGCAGTCG-3’ and 5’-CAAAATGTCTTTATTTTCACACTCAA-3’ each, 0.04 μl GoTaq-polymerase and 5,16 μl nuclease-free water. One μl of the isolated DNA was added. The PCR program was 2 min initial denaturation at 96°C, followed by 39 cycles of 30-sec denaturation at 96°C, 30-sec annealing at 56°C, and elongation at 72°C for 1 min. The program was finished by a final elongation step at 72°C for 2 min. For visualization, the samples were run on Gel-red containing 1.5% agarose gels for 20 minutes at 90 V. The results indicated if the analyzed animal was genetically male or female. Males had only one quick-running band, whereas females had two additional slower-running bands that appeared sometimes fused to one. To verify the sensitivity and specificity of the test, we took small tissue samples (with scalpel and forceps) of adult female and male salmon lice, which could be sexed by eye. The tissue samples underwent the same treatment as the larvae (DNA isolation, PCR, agarose gel). As expected, the male tissue samples yielded only one band in contrast to the female tissue samples yielding two or three bands.

### Early developmental speed

To determine whether the developmental speed of female and male salmon lice differs already in the larval stages, we placed individual salmon lice egg strings into hatching wells and monitored them closely from hatching until molting to the copepodid stage. The hatching wells were checked for the presence of copepodids several times a day (nauplii and copepodids are easily discriminable by the eye). The molting progress of lice from one egg string took several hours. Therefore, we could take samples when different proportions of the animals had already molted. Early during the molting progress, only a few copepodids and many nauplii were present in the hatching well; later, all animals had become copepodids. We harvested the samples at different time points during the population molting progress by putting the animals into 70% ethanol, thereby stopping further molting. Then, we counted the number of nauplii and copepodids under a dissection microscope. Thereby we could calculate the population’s molting progress, defined as the number of copepodids divided by the total number of animals in one hatching well. For each egg string, 24 nauplii and 24 copepodids were randomly chosen and sexed using the described PCR-based sex-determination method. Early and late during the molting progress, less than 24 copepodids and nauplii were available, respectively. In these cases (3/17 time points), all available animals of the respective stage were sexed, giving a somewhat smaller data basis for calculating the sex ratio. For each time point, we calculated the sex ratio within the nauplii and the copepodid subpopulation individually. We then calculated sex-specific ratios of animals having molted to the copepodid stage as a function of the overall population molting progress, taking the overall sex ratio within the entire population (50/50) into account.

To analyze the general sex ratio within egg string pairs, we separated three pairs into individual hatching wells and allowed them to hatch. Every hatched individual underwent DNA extraction and sex determination subsequently.

### RNA-Seq

To understand the molecular basis for differences in male and female nauplius II, we used RNA sequencing. To get enough sample material, we decided to pool several animals of the same sex. RNA was isolated from individual nauplius II animals 58–64 hours post-hatching when the animals should be in the middle of the stage. By choosing this time point, we assumed to see mainly differences directly related to sex and not to differential developmental speed. For RNA isolation, we used the Direct-Zol RNA Microprep kit (Zymo) in combination with TRI reagent (Sigma), following the manufacturer’s instructions. Elution was performed with 13.2 μl nuclease-free water. One μl of each RNA sample was reverse-transcribed with the AffinityScript QPCR cDNA Synthesis Kit (Agilent), and the cDNA diluted 1:5. Each cDNA was then evaluated by qRT-PCRs using PowerUp SYBR Green Master Mix (Thermo Fisher Scientific), with cycling parameters: initiation, 50°C for 2 min; holding, 95°C for 2 min; 40 cycles of 95°C for 15 seconds; and then 60°C for 1 min on QuantStudio 3 qPCR machines (Applied Biosystems). The expression of three genes was measured in each sample as the threshold cycle (C_T_-value), which represents the number of the amplification cycle in which the fluorescence signal exceeds the background: elongation factor 1a (*EF1A*) as a reference gene and the female-specific variants of *phb2* (5’-TGATTAGATTGTGGTGATATTCGGTA-3’, 5’-TGAGACTCAGAGAAAGACCAGCTT-3’) and *ksr2* (5’-CAGCCTTCACTAGCCCAGGA-3’, 5’-CACACTTGGCGGGTTTGAG-3’). Only samples with a C_T_ value for *EF1A* between 18 and 24, as an indicator of high-quality RNA, were used. Samples were considered female if the C_T_ value for the female-specific variant of *phb2* was between 5 and 10 units higher than the *EF1A* C_T_ value, and the female-specific *ksr2* C_T_ was between 6 and 12 units higher. Samples for which these conditions did not apply were considered males. Roughly 30 samples were combined for each pool. Overall, we created six pools, three pools per sex. Samples were quality controlled on an Agilent Bioanalyzer and used for Illumina sequencing at the University of Bergen’s Genomics Core facility.

For the analysis, fastq files were quality controlled (MultiQC, [19]) and trimmed by ten bases at the 5’ end and by four bases at the 3’ end (Trimmomatic, [20]). Reads were then pseudomapped to the public *L*. *salmonis* transcriptome (predicted transcripts based on the genome LSalAtl2s, available at Ensembl Metazoa) using Salmon [21]. The resulting counts were used for a differential gene expression analysis using DESeq2 [22]. The sex of the lice pools and their originating egg string were used as independent variables in the model, including the egg string as a batch effect. An adjusted p-value of 0.05 was used as the significance threshold, and additionally, we decided only to report genes that showed a fold change of at least 1.5 in either direction. The corresponding script is given in S1 Script.

Further analyses were performed on the public usegalaxy.eu web server [23], which offers a graphical user interface for many bioinformatical tools. The trimmed reads were mapped against the *L*. *salmonis* transcriptome using bowtie2 [24] at default settings. Additionally, the reads were mapped against the *L*. *salmonis* genome (LSalAtl2s) with STAR aligner [25]. The resulting alignment files were later opened in the Integrative Genomics Viewer IGV [26] together with the transcriptome and genome to see the mapping quality and sequences of the mapped reads at specific genomic locations.

All original fastq-files were uploaded to NCBI into Bioproject PRJNA602401.

As a follow-up of the RNA-Seq-results, we analyzed the expression of several differentially expressed genes (DEGs). To that end, nauplius II larvae from one pair of egg strings were sampled at different time points in RNAlater. Samples were taken 25, 31, 48, 55, 72, 80, 87, 102 and 106 hours post-hatching (hph). At the last time point (106 hph), the animals had already molted to copepodids. RNA from these samples was isolated individually according to the protocol used for the RNA-seq samples, with an elution volume of 8 μl. The sex was determined by the female-specific *phb2*-assay as described before. The expression of the GOIs was analyzed by qPCR in relation to the animal’s age and sex. *Ef1A* and ADT3 were used as reference genes; both genes are well-established reference genes in salmon lice [27, 28]. The qPCRs were performed as described above, and the primers used are given in S1 Table.

### Finding sex-specific SNPs

To identify sex-specific SNPs suggesting additional sex-specific gene variants, bam-files from the bowtie2-transcriptome alignment were used to call all variants using samtools [29] (samtools mpileup). Variant detection was performed using VarScan [30]. The resulting vcf file was filtered for variants with precisely three out of six heterozygous samples (HET = 3) and then filtered for variants for which only the females were heterozygous by searching for the corresponding lines in a text editor using regular expressions. Afterward, the number of female-specific heterozygous SNPs was counted for each gene in Microsoft Excel.

The same workflow was used to find sex-specific SNPs in the datasets of a previous study [11]. In this work, samples were classified based on their instar age (“young”, “middle”, “old”), representing the time since their last molt. For this article, samples of the “middle”- stage of preadult I females (Sequence Read Archive accessions SRR6179441, SRR6179443, SRR6179445) and preadult I males (SRR6179491, SRR6179446, SRR6179496) were analyzed. The complete workflow was performed on the public usegalaxy.eu web server [23].

Based on the found sex-specific variants, we identified and characterized an additional gene with the highest number of sex-specific SNPs. Sex-specific primers for PCR were deduced based on the SNPs and further sequence comparisons within the genome.

## Results

### Sex-specific gene variants

**Fig 1:**
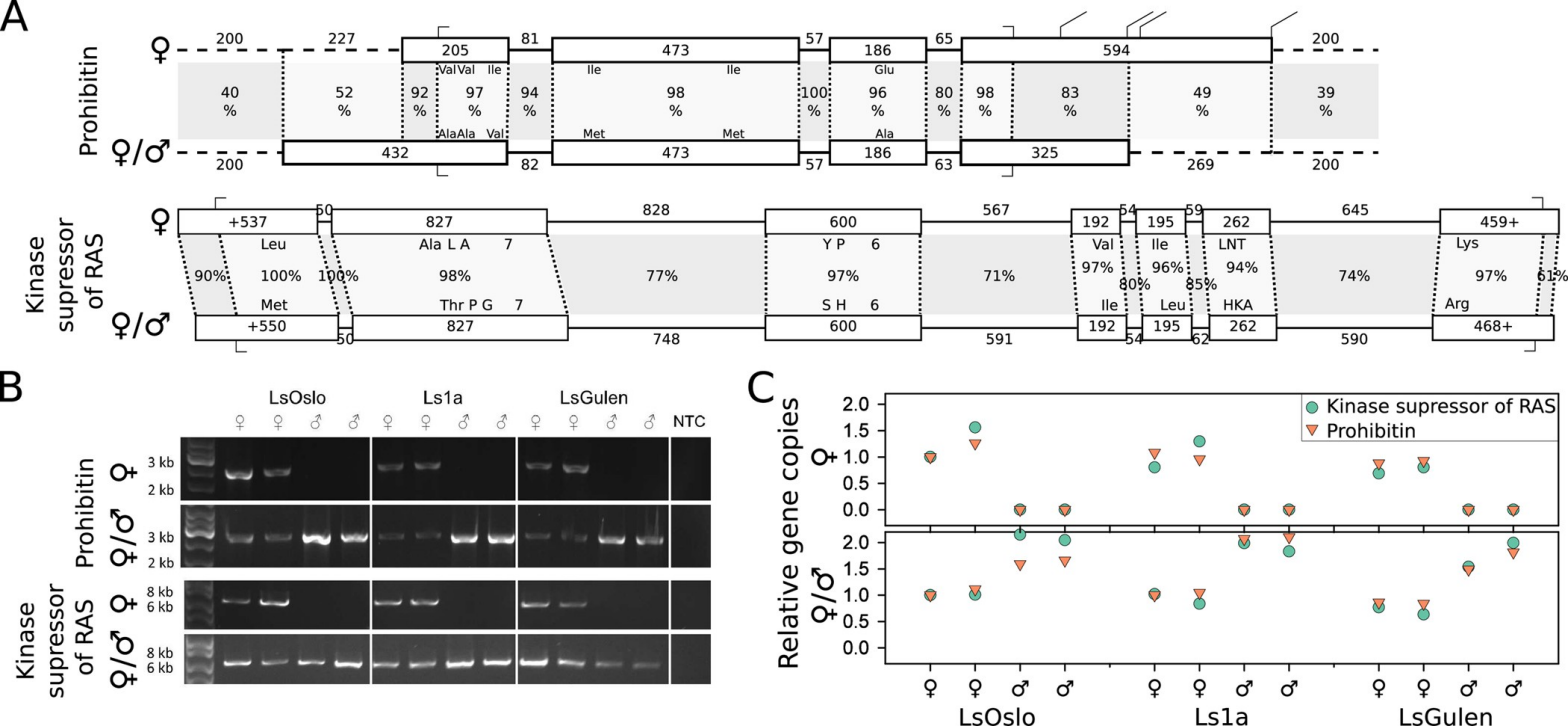
Sex-specific gene variants. A. Exon-intron-structure of the female-specific and the unisex variants of prohibitin-2 and kinase suppressor of Ras 2. Boxes mark exons, lines introns. Sizes of the structures represent the number of basepairs given over (intron) or in (exon) the structure. Shadowed areas between the two variants have been aligned and the identity (%) calculated (numbers in the middle). Amino acid changes are shown by the respective (one-letter) amino acid code at the respective sites in the exons. In the case of a high number of amino acid changes in one region, only the number of amino acid changes at that site is given. Dashed lines represent regions upstream and downstream of the UTRs. Start and Stop codons are marked. B. Presence of the female-specific and the unisex variants of prohibitin-2 and kinase suppressor of Ras 2 in the genome of males and females of different salmon lice strains as determined by PCR. C. Relative gene copy numbers of the female-specific and the unisex variants of prohibitin-2 and kinase suppressor of Ras 2 in the genome of males and females of different salmon louse strains as determined by qPCR.

We identified sex-specific variants of a *phb2* gene and a kinase suppressor of Ras 2 (*ksr2*) gene (Fig 1). PCR on genomic DNA showed that there was one variant yielding a band only in females and another variant yielding bands in males and females for both genes. From here on, we refer to these variants as female-specific and unisex variants. This finding was reproducible and consistent in all three examined salmon louse strains. Quantitative PCR on genomic DNA confirmed the absence of the female-specific variant in males. Further, it showed that males have a twice as high copy number of the unisex variants of *phb2* and *ksr2* compared to females. At a structural level, the female-specific and the unisex *phb2* variant show a high degree of similarity. In the coding domain, the exons have a sequence identity of 96–98%. Also, the introns between the coding exons are highly similar, with identities between 80 and 94%. The encoded proteins have the same length of 297 amino acids but differ in six amino acids. We found four alternative polyadenylation sites for the female-specific variant but only one for the sex-independent variant. Additionally, the 5’ UTR of the unisex variant was 227 base pairs longer than the 5’ UTR of the female-specific variant. There was little similarity between the sequences in the genomic regions up and downstream of both genes (39–40%).

The findings for *ksr2* were overall similar. The conservation of the exons was on a similar level (94–100%), while the conservation of the introns was somewhat lower. While the first intron showed 100% identity, sequence identity for the other introns was only between 71 and 85%.

The sequences have been deposited in GenBank (MW965436-MW965439).

### Early developmental speed

**Fig 2:**
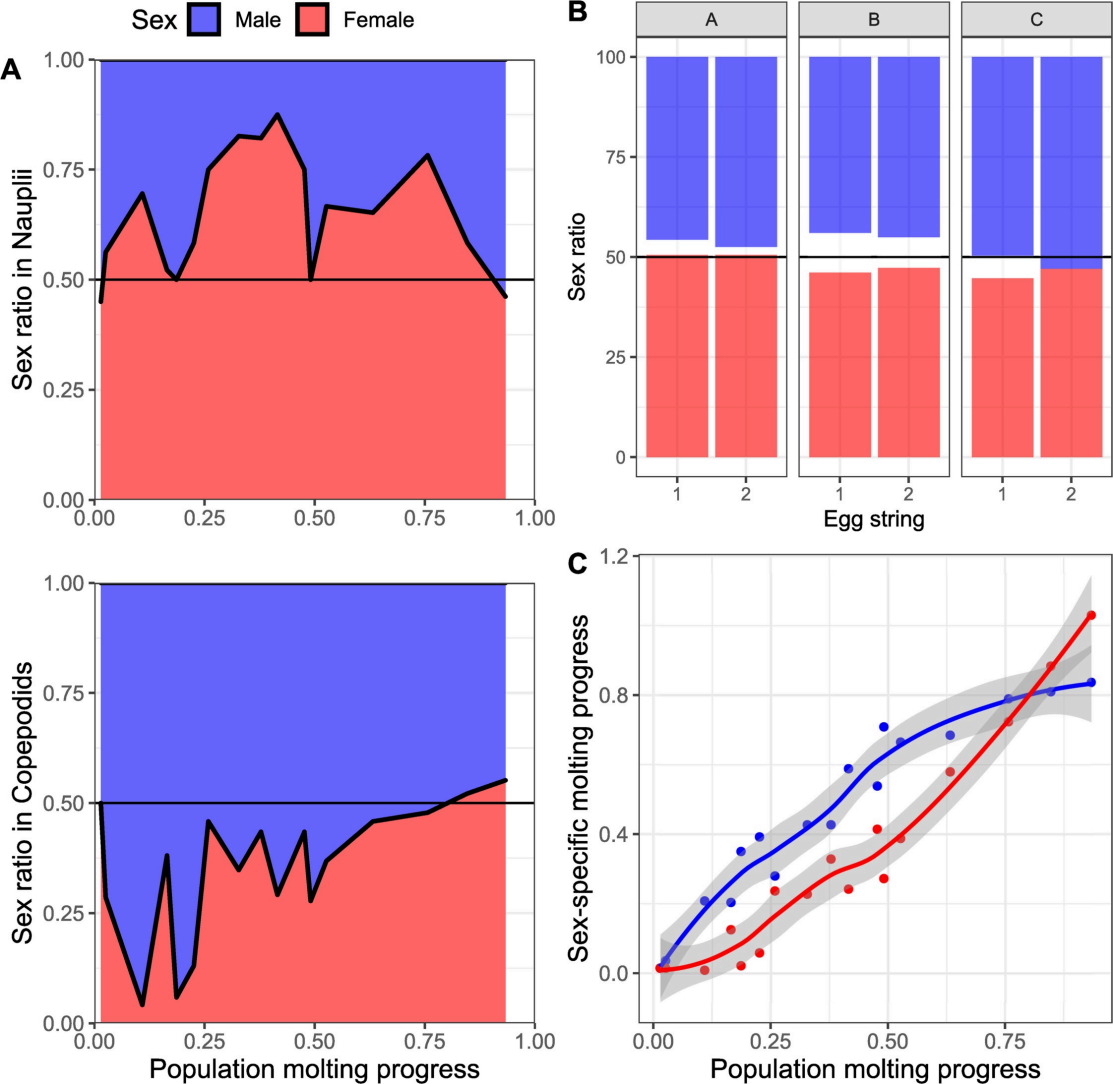
Early-stage sex ratios and sex-specific molting speed. A. Several populations descending from one egg string pair each were sampled at different time points during the molting progress from nauplius II to copepodid. The population molting progress refers to the ratio of animals of the analyzed egg string that had undergone molting to the copepodid stage at sampling. The sex ratio of the nauplius- and copepodid-subpopulations were determined individually in random samples from the population. B. The general sex ratio in the offspring from egg strings was determined at the copepodid stage. Every hatched animal was sexed by PCR. The white areas show animals for which the sex determination was not successful. C. Calculated sex-specific molting progress. Based on the data from A and B, we calculated the sex-specific molting progress, representing the rate of males and females having undergone molting to the copepodid stage relative to the overall population molting progress, respectively.

We developed a sex-determination PCR assay utilizing genomic DNA and the *phb2* gene (see Methods part). This assay could be adopted for a high-throughput format in 96-well plates. With use of this assay, we were able to determine the development of the sex-ratios of the nauplius II and copepodid subpopulations during the transition of the whole population from nauplius II to copepodid (Fig 2). Additionally, we could determine the general sex ratio of the offspring of individual salmon lice mothers. The general sex ratio of the egg string offspring was close to 1:1, although it was slightly differing between tested egg strings. The sex ratios of offspring from each egg string of the pairs were highly similar. However, we found a difference in the timing of molting from nauplius II to copepodids between females and males. During molting of the population, lasting several hours, the ratio of females in the not-yet-molted nauplii was generally over 50%, with a maximum of 88% females when 41% of the total population had molted. In the already-molted-copepodids, there were generally more males, with a ratio of 95%, when 10% of the populations had molted. These differences in sex composition disappeared when the population’s molting progress reached its end.

Based on these data, it became apparent that the molting of male nauplius II began on average earlier than the molting of the female lice (Fig 2C). When ca. 20% of the animals had molted, male and female molting speeds converged. After 60% of the population had molted, the remaining female nauplii molted faster than the males.

### Early differential gene expression

To validate that the pooling of the animals into female and male pools was successful, we mapped the reads to the female-specific and unisex variant of *phb2* and *ksr2* using bowtie2. In the case of the female-specific gene variants, there was more coverage in the female pools than in the male pools (S1 Fig). In the case of *phb2*, applying a filter for mapping quality of 10 (thereby removing reads that mapped more than once) completely removed all mapped reads from the male library (S1 Fig). On the other hand, both libraries produced a high coverage, independent of sex, on the unisex gene variants. These findings suggest a successful separation into female and male animal pools.

**Fig 3:**
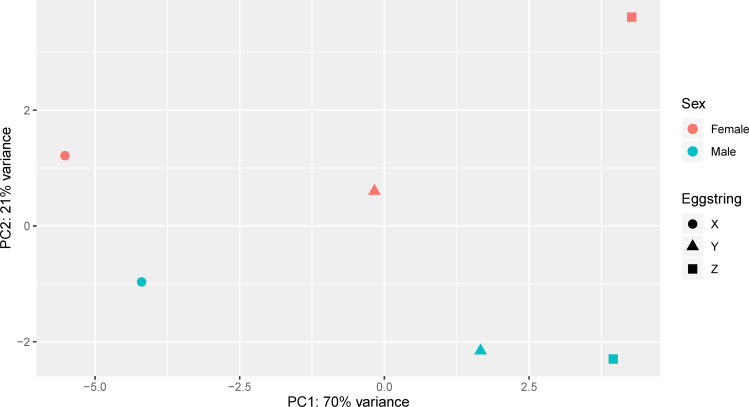
Principal component analysis of the general gene expression in female and male nauplius II *L*. *salmonis* samples. Animals from three different egg strings (X, Y, Z) were sampled in the nauplius II stage, and the sex of the individual animals was determined by qPCR. Males and females from each egg string were pooled together, respectively. RNA was isolated and employed in RNA-Seq.

A principal component analysis of the samples (Fig 3) revealed that most of the variance (70%) could be explained by the origin of different egg strings. On the contrary, salmon lice sex only explained a comparably small part of the variance (21%). In concordance, a low number of genes was differentially expressed between the sexes. Only twenty genes were significantly upregulated over 2-fold in females and just five in males (S2 Fig and S2 Table). Setting the filter less strict to a |FC|>1.5 (up- or downregulation with a fold change (FC) of at least 1.5), the number of female-biased and male-biased genes was the same (25 in each). Not filtering by FC, only taking the adjusted p-values (padj) into account yielded 191 DEGs. EMLSAG00000000026 was the gene with the highest male bias, with a 5.2-fold higher expression in males than in females (padj<0.001). This gene encodes a protein containing a DnaJ-domain typical for HSP40-proteins. While the second highest expressed sex-biased gene (EMLSAG00000003593) does not encode known domains, the third gene (EMLSAG00000003353) encodes an insect cuticle protein. Two additional genes encoding insect cuticle proteins, EMLSAG00000004219 and EMLSAG00000006452, were upregulated in males as well. For females, several genes with a higher expression level compared to the males were identified. Most of these were encoding proteins of unknown function, but some were annotated as functional proteins like a mitogen-activated protein kinase kinase kinase (MAPKKK, EMLSAG00000003032), a cyclin (EMLSAG00000004707) or the transcription factor E2F2 (EMLSAG00000007877).

### Sex-specific gene expression patterns during the nauplius II stage

**Fig 4:**
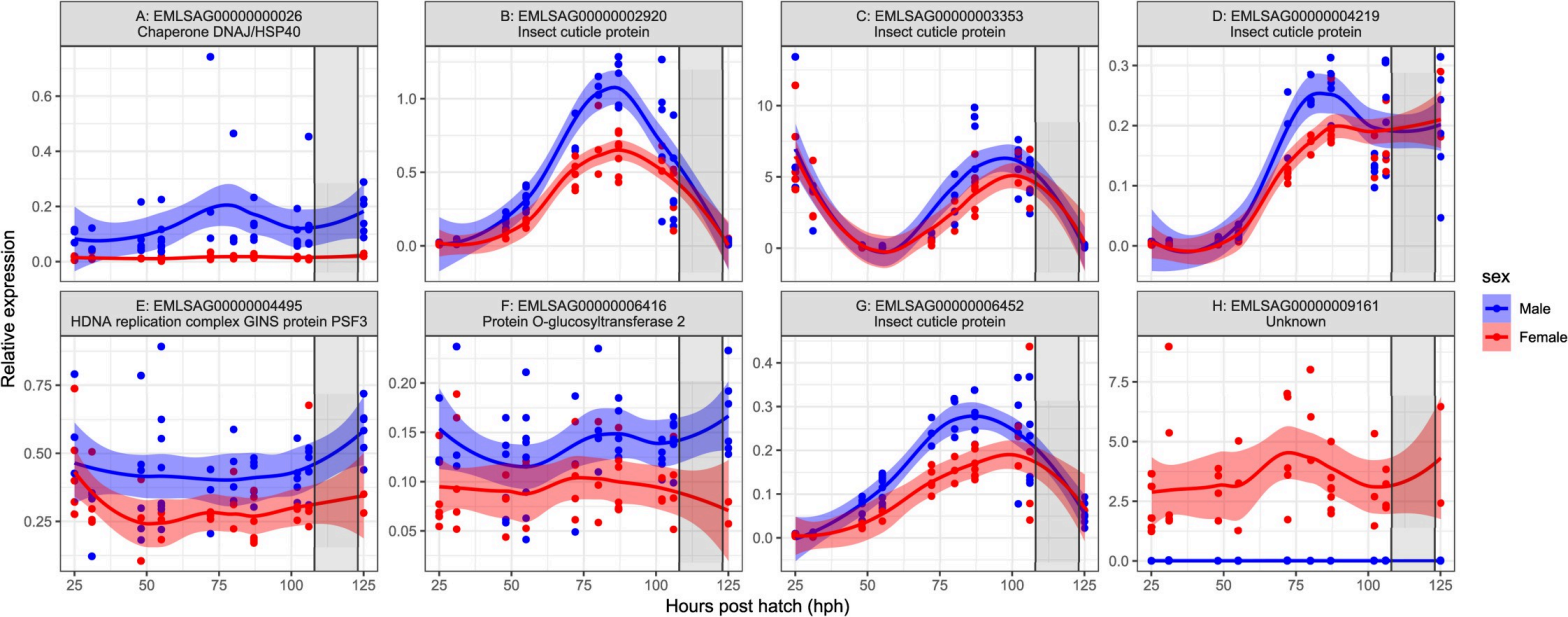
Expression of selected genes of male and female individual salmon lice during the nauplius II stage. Offspring from one egg string pair was sampled at different time points during development. The sex of every animal was determined, and several target genes were measured using qPCRs. Sampling started during the nauplius II stage and stopped in the copepodid stage. The grey rectangle marks the time frame in which molting from nauplius II to copepodid has taken place.

To validate our findings from RNA-Seq-data, we measured several of the differently expressed genes via qPCR on additional samples from another egg string pair. As temporal effects during development might affect gene expression levels, we decided to sample salmon lice larvae (from one egg string pair) at several time points during development: from the onset of the nauplius II stage until molting to the copepodid stage. Overall, the results from the RNA-Seq analysis were confirmed (Fig 4). EMLSAG00000000026 (A) was stronger expressed in males than in females at all time points. EMLSAG00000009161 (H), on the other hand, was stronger expressed in females than in males. We also applied the primers targeting EMLSAG00000009161 on genomic DNA and obtained bands from males and females (S3 Fig). The expression for these two genes was relatively stable through the investigated early developmental stages (Fig 4A and 4H), whereas the expression for the insect cuticle proteins homologs changed during the development in specific ways (Fig 4B–4D and 4G). EMLSAG00000002920 (B) expression peaked several hours before molting to the copepodid stage, whereas EMLSAG00000003353 (C) started on a high level directly after the molt, then decreased, just to increase again briefly before molting. In the copepodid stage, the levels were very low. EMLSAG00000004219 (D) started low, then increased in the middle of the stage and remained stable up to the copepodid stage. EMLSAG00000006452 (G) started low, reached its maximum in the middle of the nauplius II stage, and then decreased again until a new low in the copepodid stage. The expression patterns of the genes encoding insect cuticle proteins were partly overlapping, partly differing between males and females. For EMLSAG00000002920 (B), the peak was almost twice as high for males as for females. This was also the case for EMLSAG00000006452 (G). For EMLSAG00000004219 (D) and EMLSAG00000003353 (C) the male and female expression was quite alike. Two other genes, encoding GINS protein PSF3 (EMLSAG00000004495; E) and a protein O-glucosyltransferase 2 (EMLSAG00000006416; F), were consistently higher expressed in males than in females.

### Sex-specific heterozygous SNPs

We searched in the RNA-Seq data for genes that showed multiple female-specific heterozygous SNPs within their sequences after mapping (Table 1). Thirteen genes with at least ten such positions were identified. Additionally, we analyzed publicly available samples from the preadult I stage with the same workflow and compared the results. Ten out of the 13 genes identified in nauplius II larvae also showed female-specific heterozygous SNPs in preadult I animals. The number of bases considered heterozygous for these genes was generally lower in preadults than in nauplii.

**Table 1:** Transcripts with more than 10 heterozygous positions in females after mapping.

Transcript	SmartBlast hit	Nau II (this study)		Preadult I (previous study^a^)	
		Ranking	Fem-spec Heterozygous positions	Ranking	Fem-spec Heterozygous positions
EMLSAT00000007877	transcription factor E2F2	1	42	9	13
EMLSAT00000003771	Ecdysone-induced protein E75a	2	31	1	40
EMLSAT00000008398	transcription initiation factor TFIID subunit 8	3	25	5	17
EMLSAT00000010583	sprouty-related	4	23	2	20
EMLSAT00000010156	?	5	21	21	7
EMLSAT00000010095	Prohibitin-2	6	20	3	20
EMLSAT00000003780	DNA topoisomerase 3	7	18	-	0
EMLSAT00000004707	G1/S-specific cyclin-E1	8	17	11	13
EMLSAT00000002851	Two pore potassium channel protein sup-9	9	16	-	0
EMLSAT00000008926	?	10	14	-	0
EMLSAT00000004632	Proteasome subunit beta	11	14	6	14
EMLSAT00000004002	PHD finger protein 14	12	13	12	11
EMLSAT00000003836	?	13	10	17	7

^a^ Reanalysed data from [11].

When comparing the female-biased expressed genes with the list of female-specific SNPs, we found a quite high overlap of these lists. More than half (14/25) of the DEGs had at least one female-specific SNP.

When we searched for male-specific SNPs in the same way, the maximum number of SNPs per gene was three, and a further investigation of the mapping to the corresponding genes in the integrated genome viewer (IGV) showed that this was due to an artifact in the algorithm instead of a real heterozygous male-specific SNP.

**Fig 5:**
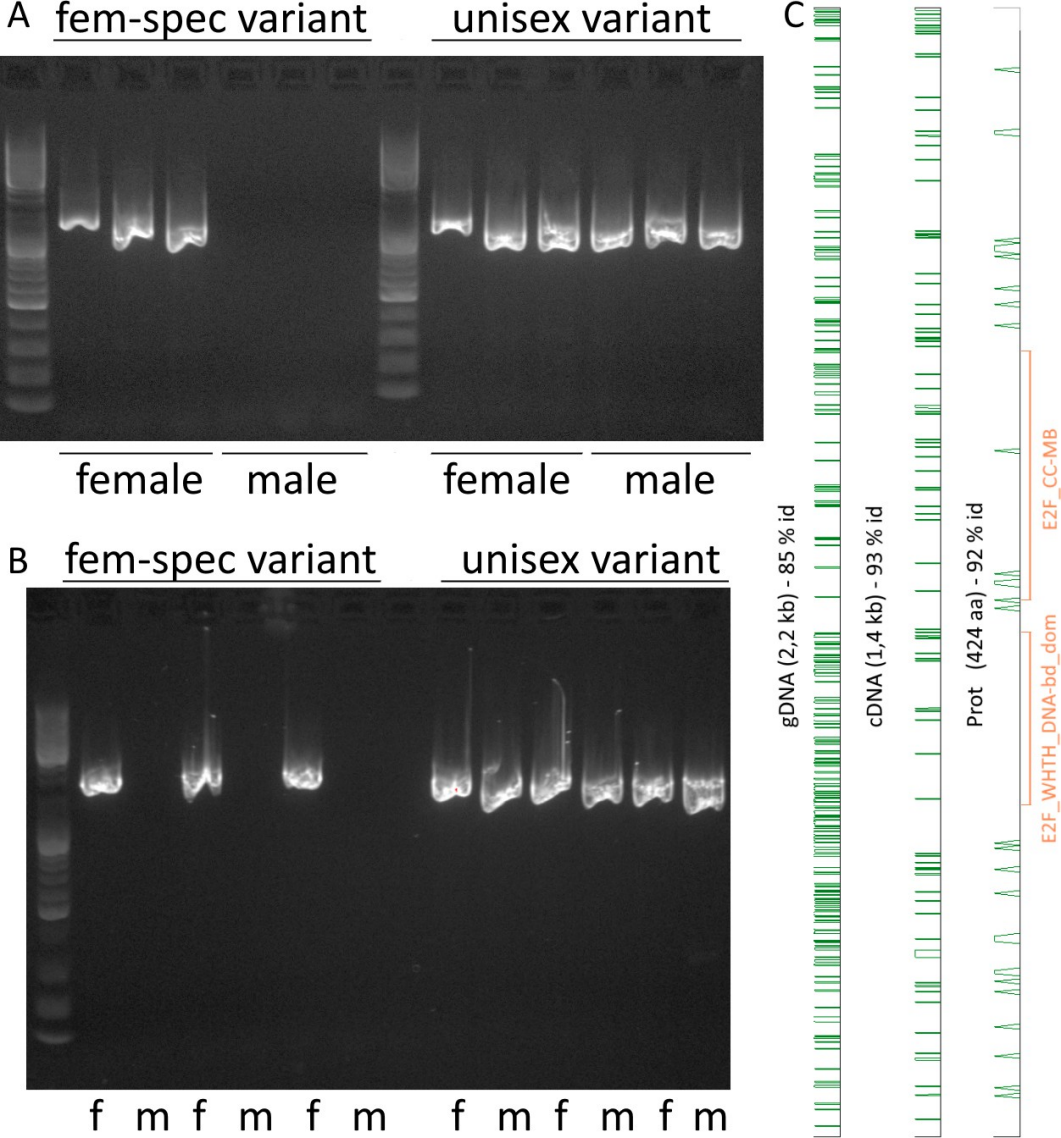
Sex-specific variants of E2F transcription factor EMLSAG00000007877. Primers targeting the CDS of the female-specific variant and the unisex variant were used in PCR of female (f) and male (m) samples. A. cDNA. B. genomic DNA. C. Alignment of the two variants on gDNA, cDNA, and deduced protein level. 5’-end/N-terminus are at the bottom of the graphs. Green marks mark differences between the female-specific and unisex variants. Conserved Interpro-domains are marked in orange in the protein lane.

To assess if the accumulation of female-specific heterozygous SNPs in one gene indicates a gonosomal location of the respective gene, we designed variant-specific primers for the gene with the highest number of such SNPs (EMLSAT00000007877), which encodes a transcription factor. The primers targeting the female-specific variant produced a band in gel electrophoresis only when female genomic DNA was used as a template in the PCR reaction, whereas primers targeting the other variant gave bands with genomic DNA from both sexes (Fig 5).

EMLSAT00000007877 encodes a member of the E2F transcription factor family. We found a high number of differences (15%) on a genomic level between the female-specific variant and the unisex variant, 7% differences on a cDNA level, and 8% differences on protein level. The amino acid exchanges do not seem to be randomly distributed but are somewhat concentrated at the N-terminal end of the protein (see Fig 5C). Comparing the alignment to results from an Interpro analysis, no differences in the “E2F/DP family, winged-helix DNA-binding domain” were detected, but several in the “E2F transcription factor, coiled-coil (CC)—marked box (MB) domain”. Among these, two mutations were located in the predicted heterodimer interface. The sex-specific sequences have been deposited in GenBank (MW965440, MW965441).

## Discussion

### Salmon lice have a ZW sex-determining system

Our results showed that salmon louse sex is determined based on a ZW sex-determining system which is in agreement with a recent analysis of the salmon louse genome [31]. Such a system has been suggested before based on one SNP in the prohibitin-2 gene [12] and on several SNPs in several other genes [13], among them the kinase suppressor of Ras 2. However, by sequencing two complete sex-specific genes, we could provide additional proof that both *phb2* and *ksr2* exist in two different variants. While the protein sequences of *phb2* and *ksr2* are conserved, intronic regions, especially for the *ksr2* gene, are far more different. One gene variant is only present in females, whereas the other variant is present in both sexes. Quantitative PCR on genomic DNA targeting the female-specific and unisex variants, respectively, showed about twice as high a genomic copy number of the unisex variant in males compared to females. This finding is in agreement with a ZW sex-determining system where both female-specific genes are assumed to be located on the W chromosome and the unisex variant on the Z chromosome. Female lice are then heterogametic (ZW), whereas male lice are homogametic (ZZ).

Interestingly, no sex-specific SNP has so far been found in the *phb2* gene of *Caligus rogercresseyi*, another sea louse species [32]. However, only cDNA sequences were analyzed in that study, and examining the intronic sequences would add confidence to that conclusion due to the potential accumulation of intronic silent mutations at a higher rate. Alternatively, the sex-determining systems might have evolved in a different way between these two Caligidae species.

Generally, not all copepods have a genetic sex-determination system. In at least four genera of free-living copepods and three parasitic species, environmental sex determination has been described [33]. Primarily temperature affects sex in several species. A genetic and environmental sex determination system can also be combined: In *Cyclop viridis*, sex is determined genetically by sex chromosomes, with heterogametic females [34]. Nevertheless, the sex ratio is affected by temperature increase or UV exposure during egg deposition in this species. Some copepod species even display sex change and intersexuality [35]. *Tigriopus californicus* does not possess heteromorphic sex chromosomes, but several genes control sex determination in a polygenic sex-determination system, also influenced by the environment [36].

Although not explicitly searching for it, our experiments seem not to hint at any additional effects on sex determination apart from the genetic factors. In all cases, when analyzing adult animals, phenotypic and genotypic sex-markers were in correspondence. We did not perform the experiments under different environmental conditions. Therefore, we cannot exclude the possibility that the environment might have a particular effect; however, this likely would have been observed before.

A genetic sex-determination system is also well in line with the approximately 1:1 sex ratio in larvae obtained from egg strings in this study. To our knowledge, this is the first report of sex ratios measured at the egg-string level.

We performed the determination of the primary sex ratio directly after egg string hatching under standardized laboratory conditions. This suggests a regular random distribution of the sex chromosomes to the offspring. Nevertheless, a balanced primary sex rate does not necessarily indicate an equally balanced sex ratio in the later stages. Possible factors contributing to differences in the sex ratio might be differences in longevity of the sexes. Also, behavioral differences can come into play. In the case of salmon lice, females can produce a high number of egg strings for months after just one copulation, whereas the male lice can fertilize several females, which might lead to a more risk-prone behavior to find a new partner. It has been shown that adult male salmon lice are more mobile and transfer more frequently between hosts than females [37]. As changing hosts can be dangerous, this kind of risky behavior might change the sex ratio. The lice loss rate of males might also depend on the host size [38]. Related to aquaculture practices, the use of cleaner fish might also shift sex ratios. Because adult female salmon lice are significantly larger than male ones, they might be easier detected by the cleaner fish and preferably eaten. As the susceptibility to emamectin benzoate is sex-dependent [39], usage of this drug might influence the sex ratio of a salmon louse population.

Norwegian law demands that salmon louse numbers are collected in aquaculture regularly [40]; however, these numbers cannot be used to deduce the sex ratio of the population. Adult female lice are counted separately, while adult males, preadult males, and preadult females are collectively classified as “mobile stages.” [41] analyzed six studies reporting male and female counts from wild and farmed Atlantic salmon and they could not find a generally valid sex ratio. In contrast, they concluded that host population, parasite population, and environment might influence the sex ratio of salmon lice [41]. Nevertheless, there seems to be a tendency to female-biased populations in the sea and male-biased populations in aquaculture [38].

The identification of sex-specific variants with several closely located SNPs enabled the development of a molecular sex test. We have developed two tests: a test based on the C_T_ values from qPCR using cDNA from the test specimens and a test using PCR on genomic DNA followed by agarose gel electrophoresis. Both tests can successfully be conducted on the early nauplius I stage. Until now, only later developmental stages could be sexed. From the preadult I stage, the sexes are easily recognized to the naked eye, but for chalimus II, several measurements are required to identify the sex of a specimen [5]. Determining chalimus II sex through measurements is very time-consuming [14], but the methods developed in the present study significantly reduce the effort and open up new experiments where sex in the early stages can be identified.

For the planktonic stages of salmon lice in the field, no data on sex ratios are available, likely due to the lack of a specific sex marker. The findings from this study might change that. Additionally, due to the low abundance of salmon lice larvae in zooplankton samples, their identification has been challenging so far [42]. The development of new methods employing species-specific fluorescence signals, depending on the fixation of the samples in formalin, can simplify this process [42]. Recently, a method for isolating DNA from formalin-fixed plankton samples has been presented [43], eliminating problems connected to the crosslinking of DNA and proteins by formalin for molecular biology applications. Combining all three methods: identifying salmon lice larvae with fluorescence, DNA extraction from formalin-fixed samples, and the sex-determination assay described in this article might deliver new knowledge about sex ratios of salmon lice larvae in the oceans.

### Males develop faster than females at an early stage

The present study showed that male nauplius II larvae molt on average earlier than female nauplius II larvae to the copepodid stage. This is similar to the later developmental stages. From the chalimus II stage and until the adult stage, males develop significantly faster than females [14]. However, this is the first time that it has been demonstrated that the sexual difference in growth rate is established early at the free-living stages (maybe in the embryo also) and could be a feature throughout the life cycle. This indicates that there is a molecular mechanism that induces a higher developmental speed in males than females. However, a potential differential developmental speed for the time from hatching until the first molt (nauplius I -> II) might be very difficult to prove, as there are few visible morphological differences between the nauplius I and II stage. Additionally, the duration of the life stage is overall short (ca 24 h at 10°C), and no molecular markers to distinguish between these stages have been identified yet.

When considering the causes for the identified differences in developmental speed, one must distinguish between proximate and ultimate causes. Unfortunately, our experiments cannot entirely explain either of these, but allow for some speculations.

We had hoped to find the proximate causes for differential speed, the mechanisms behind, by performing RNA sequencing. We expected that there might be crucial differences in gene expression between male and female nauplius II larvae. By analyzing the functions of the differently expressed genes, we hoped to find clues for the molecular mechanisms behind the observed differences in developmental speeds. However, only a few genes were differentially expressed, and neither of them could provide an apparent explanation for the developmental speed variations. Nevertheless, even small, undetected gene expression changes might have a severe biological impact. Instead of major expression differences, we found a number of SNPs, which affect the amino acid sequence of several proteins and thereby might affect their structure and function. We discuss this in more detail below. Another proximate cause can be differential hormone levels in males and females. Ecdysteroids are the hormones responsible for molting in crustaceans [44]. An increase of the molting hormone levels leads to molting; however, the mechanism by which the hormone increase is triggered is not well understood, as far as we know.

The ultimate cause of the described phenomenon might be related to intrasexual selection. Males that reach the last life stage and thereby sexual maturity earlier than their competitors have an a increased likelihood to mate with several females. This might be a significant factor, selecting for quicker development and earlier sexual maturity in males.

However, the relevance of the different developmental speeds in nature remains to be examined. While most external parameters were controlled for and stable in our lab experiments, the situation in the field is much more dynamic, e. g., water temperatures change. Further studies should show how far our observations in the lab can be validated in the field.

### Genes involved in cell cycle control have sex-specific variants

We found several genes which showed heterozygous base positions in the female samples but not in the male samples. We conclude that these genes are most likely located on the sex chromosomes. While the males carry two copies of one (the unisex) variant, the females carry two variants with one copy of each. In line with a female-specific W chromosome, only genes with female-specific but not male-specific heterozygous mappings were identified. Many of the genes for which we found heterozygous bases in our data also showed heterozygosity in a previously published salmon louse dataset [11]. The difference in the total number of heterozygous positions for the different genes between the nauplius and the preadult dataset can be explained by different gene expressions between the life-stages and the different sequencing-depth between the libraries. The library depth of the preadults was considerably lower than the nauplii libraries. Thereby the algorithm might fail to detect heterozygous positions in some genes, especially for low expressed genes. Fascinatingly, this information about sex-specific sequences has been available in the preadult dataset but without being recognized as such.

Transcription factor E2F2 (EMLSAT00000007877, 42 SNPs) was identified as the gene with most female-specific heterozygous SNPs. We confirmed by PCR that two different variants exist, and they must be located on the sex chromosomes. This suggests that our analysis overall is a reliable way to identify sex-specific SNPs. Until now, only 27 sex-specific SNPs have been described [13] in several genes, whereas we found 42 such SNPs within the E2F2 gene alone. Several of these SNPs were nonsynonymous and led to changes in the amino acid sequence. These could lead to different functions of the female-specific and unisex gene products, and it could be of particular importance since changes in a transcription factor might have consequences for the expression of downstream targets. This could also contribute to the observed differences in developmental speed between males and females. Members of the E2F transcription factor family have been called “key regulators of cell proliferation” [45]. The CC-MB domain of E2F is used for heterodimerization with the DP transcription factor. We found several amino acid exchanges in this domain in the two variants, which might suggest different heterodimerization capabilities and potentially different functions. In cell culture, it has been shown that E2F1 induces the gene expression of cyclin E [46]. G1/S-specific cyclin E1 (EMLSAG00000004707) is on our list of genes with several female-specific heterozygous SNPs and thereby probably located on the sex chromosome. Cyclin E is essential for the transition from G1- to S-phase during the cell cycle [47]. E2F is also regulated by *phb2* [48]. In the salmon louse EMLSAG00000010095, encoding *phb2*, the gene used for our sex-determination assay, is located on the sex-chromosomes and these two variants differ in six amino acids. Together, at least three genes involved in cell cycle control, which are interacting with each other, are likely located on the sex chromosomes, and this might influence different developmental rates and the later size differences of males and females. Also, in chalimus and preadult II lice, cell cycle phase transition was a GO term that was enriched for female-biased genes [11]. Unfortunately, it is challenging to experimentally address whether the SNPs in these genes are crucial for the developmental differences. RNAi has been established as a method to analyze gene functions in salmon lice [49, 50]. However, we could not find regions of any of the genes sufficiently different between the sex-specific variants to make variant-specific double-stranded RNAs. The specificity of RNAi has limitations, and off-target effects can be a problem [51]. We assume that trying to knock down, for example, the female-specific variant of one of the genes would inevitably lead to the knock-down of the other variant. Hence, more precise gene-editing methods, for example, CRISPR-based gene editing, might be a way to validate the significance and function of the different variants. However, this method has not been established in salmon lice yet and may be problematic to perform due to the small size of the animals and their eggs and difficulties in obtaining tissue samples from the lice without lethal effects.

### Few genes are differently expressed between sexes in the early life stage

Overall, only a few genes were expressed differently between male and female nauplius II larvae as measured by RNA sequencing. Of the female-biased genes, the majority (21/25) has also been identified as being differentially expressed between males and females based on RNA-Seq of chalimi and preadult animals [11]. The accordance between the male-biased genes was lower. Nine of 25 genes were not only expressed higher in male nauplius but also in chalimus and preadults suggesting stage-specificity of sex-biased gene expression. However, a cautious interpretation of the RNA-Seq results is necessary due to the high rate of genes identified as differentially expressed, for which we also found sex-specific SNPs. These genes are likely located on the sex chromosomes, which are apparently not very well represented in the current genome assembly LSalAtl2s. Some of the genes were assembled as one gene, others (partially) as two. The correct mapping of the reads is thereby challenging. This calls for further improvements of the salmon lice genome [31], which was assembled based on reads with a length between 50 and 1100 base pairs [31]. New sequencing techniques allow for read lengths of more than 10 kb [52]. Such a length should help to distinguish female-specific and unisex variants of gonosomal genes.

A thorough examination of the gene expression of some of the DEGs without sex-specific SNPs during development was performed. Data analysis showed that gene expression of some genes in salmon lice during a life stage is not stable but highly variable, as shown before [11]. Especially the insect cuticle proteins showed specific patterns, and the gene expression depended on the instar age and the GOI. Insect cuticle proteins are part of the exoskeleton of insects and other arthropods. They are known to be hormonally regulated by ecdysteroids [53] during the molting cycle. This highlights the importance of a suitable sampling regime, especially in the early life stages, where changes occur in a narrow time window. Comparisons of gene expression between treatments should always be made between samples of the same instar age [11]. Altering gene expression levels within an instar were observed during the development of chalimus I to preadult 1 [11]. Our qPCR experiment expands these findings to the nauplii and copepodid stages and underlines the general validity of these findings. Additionally, when sampling near the end of a nauplius stage, it is critical to sample before the first animals molt to the next stage since the population’s sex ratio will change and potentially influence gene expression patterns. Anyway, two of the analyzed cuticle proteins peaked at a higher expression level in males than in females, suggesting that the exoskeleton composition and structure between male and female salmon lice might already differ to a certain degree in the nauplius II stage.

One other male-biased expressed gene was EMLSAG00000000026, encoding a DnaJ domain, indicating that it is encoding an HSP40 protein. HSP40 proteins regulate the activity of HSP70 proteins and are thereby involved in protein translation and folding [54]. The heat shock protein repertoire of salmon lice and its response to different has been characterized in detail [55], but EMLSAG00000000026 was not among the analyzed genes. A reason for the upregulation of exactly one specific HSP is not apparent. Nevertheless, there are documented examples of sex-biased expression of HSPs. In rat muscle, several HSPs are stronger expressed in males than in females [56]. The HSP90A gene was higher expressed in females than in male copepods in *Eurytemora affinis* [57]. We also noticed higher levels of EMLSAG00000004495 (encoding the Psf3 subunit of the GINS complex) in males than in females. GINS is essential for DNA replication [58], and changes in replication might contribute to differences in developmental speed.

## Conclusions

This study presented additional evidence that salmon lice have a sex-determination system based on ZW-chromosomes. Females are heterogametic, whereas males are homogametic. We sequenced three genes from the proposed sex chromosomes, where one of these had not been identified to contain sex-specific SNPs previously. These sequences made it possible to develop a PCR assay to determine the sex even of the earliest life stages of the salmon louse. Using this test, we could show that male nauplius II are developing faster than females and molting into the copepodids stage earlier than the females. Despite the differential developmental speed in the nauplius II stage, only a few genes are differentially expressed between the sexes in this early stage. Additionally, we could identify sex-specific SNPs in several new genes suggesting that they are located on the sex chromosomes. Several of these genes are involved in cell cycle control and might thereby be involved in the differential developmental speed due to sex-specific mutations.

## Supporting information

S1 Fig Coverage of female-specific and unisex variants of prohibitin-2 and *ksr2* in female and male specific libraries. The reads of the female (pink) and male (blue) library from one egg string were mapped against the female-specific and unisex variants of the sex-specific genes. The dotted lines were not considering mapping quality, the non-dotted lines were filtered with a mapping quality over 10, eliminating reads mapping to several transcripts. A. female-specific variant of prohibitin-2. B. female-specific variant of *ksr2*. C. unisex variant of prohibitin-2. D. unisex variant of *ksr2*.
(PDF)

S2 Fig Heatmap of differently expressed genes with a q-value<0.05 and |FC|>2. Animals from three different egg strings (X, Y, Z) were sampled in the nauplius II stage and the sex of the individual animals was determined by qPCR. Males and females from each egg string were pooled together respectively. RNA was isolated and employed in RNA-Seq. Red colors indicate upregulation, blue colors downregulation.
(PDF)

S3 Fig Presence of EMLSAG00000009161 in genomic DNA of male and female salmon lice. EMLSAG00000009161-targeting primers were used to amplify a fragment of the gene using PCR, using genomic DNA from male and female individuals of three different salmon louse strains. The same primers had shown a highly female-biased pattern in qPCR of cDNA from both sexes.
(TIFF)

S1 Table Primers used in this study. (XLSX)

S2 Table Results of the DeSeq2 analysis. (CSV)

S1 Script R-code for the calculations. (R)

S1 Appendix Original gel pictures. (ZIP)
