# Supplementary figures and images for: Sex differences in the early life stages of the salmon louse Lepeophtheirus salmonis (Copepoda: Caligidae)
Source: PLoS One. 2022 Mar 31;17(3):e0266022. doi: 10.1371/journal.pone.0266022 (PMC8970357; doi:10.1371/journal.pone.0266022)

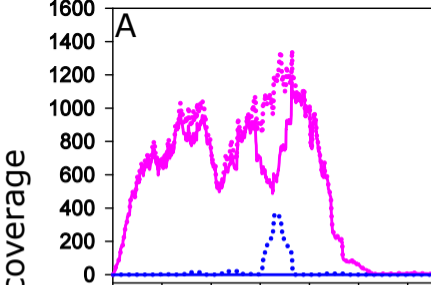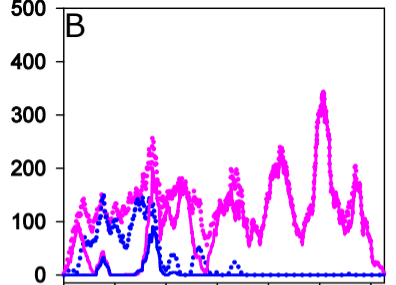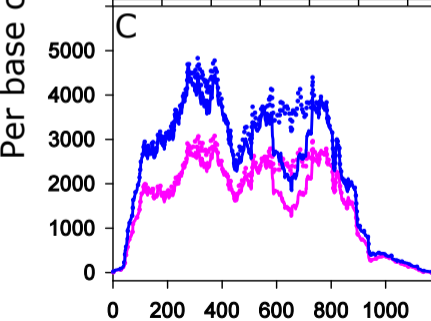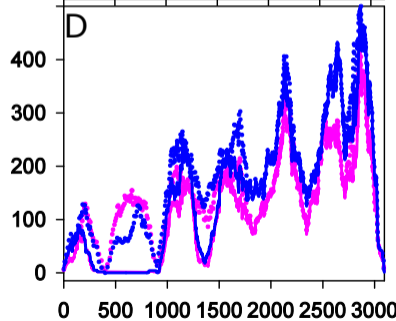

Position (bp)

Supplement: S1 Fig — The reads of the female (pink) and male (blue) library from one egg string were mapped against the female-specific and unisex variants of the sex-specific genes. The dotted lines were not considering mapping quality, the non-dotted lines were filtered with a mapping quality over 10, eliminating reads mapping to several transcripts. A. female-specific variant of prohibitin-2. B. female-specific variant of ksr2. C. unisex variant of prohibitin-2. D. unisex variant of ksr2. (PDF) [file pone.0266022.s001.pdf]

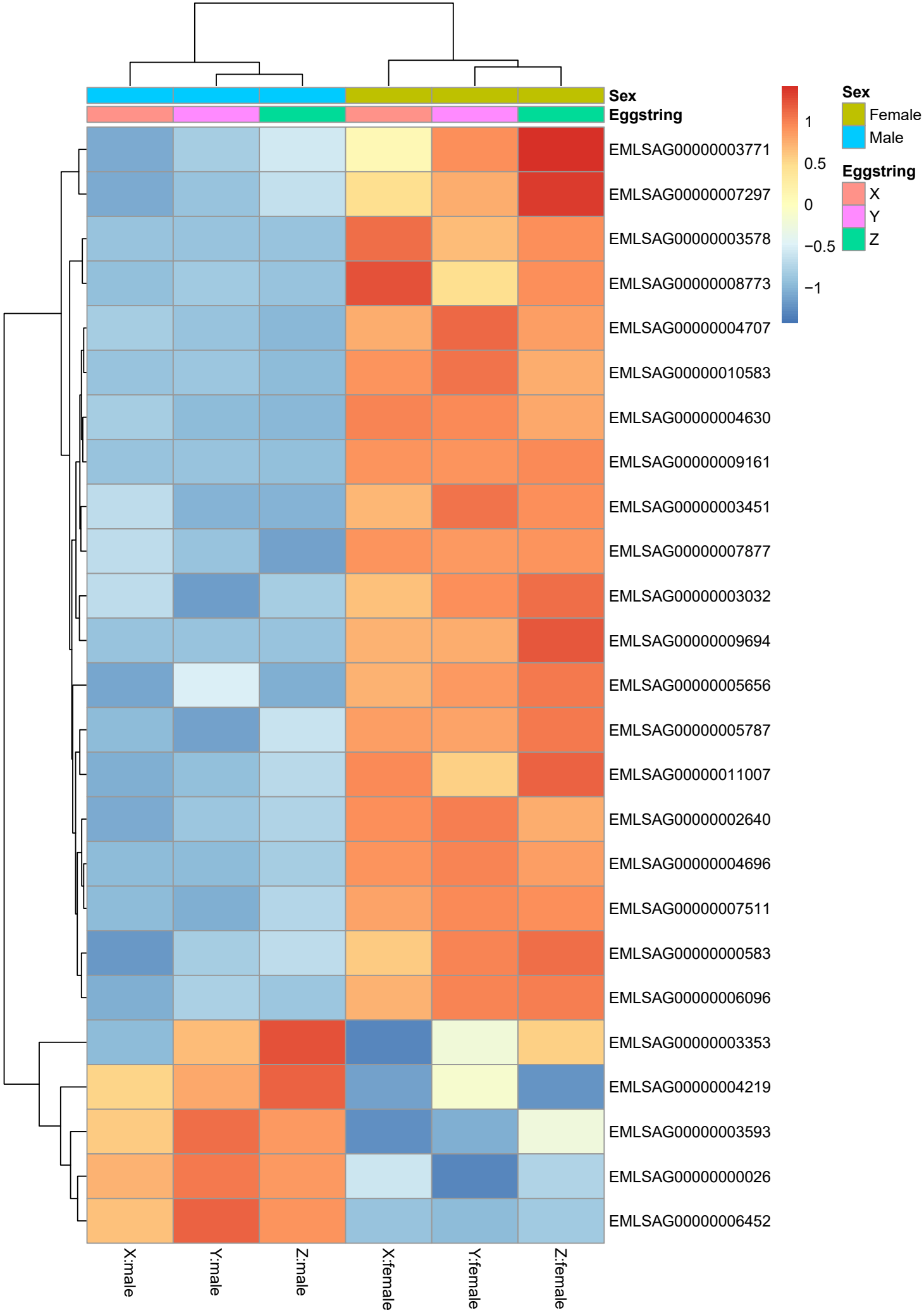

Supplement: S2 Fig — Animals from three different egg strings (X, Y, Z) were sampled in the nauplius II stage and the sex of the individual animals was determined by qPCR. Males and females from each egg string were pooled together respectively. RNA was isolated and employed in RNA-Seq. Red colors indicate upregulation, blue colors downregulation. (PDF) [file pone.0266022.s002.pdf]

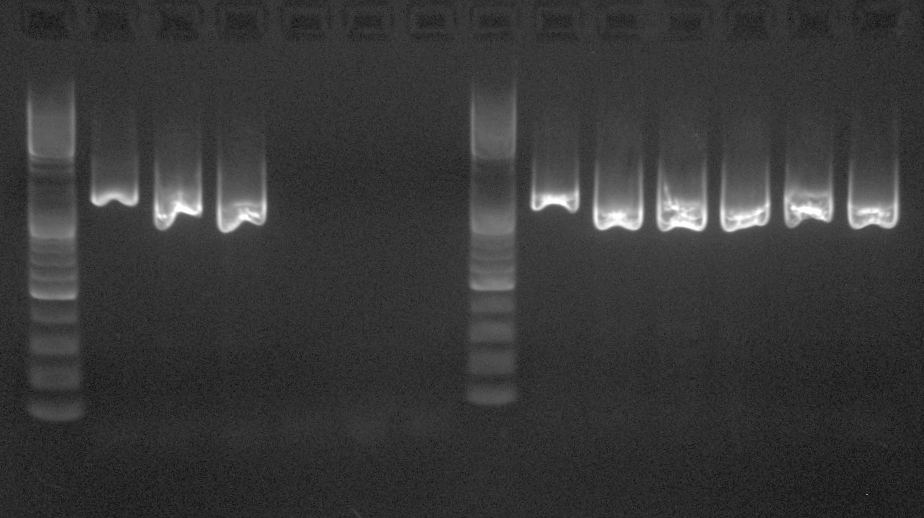

Supplement: S1 Appendix — (ZIP) [file pone.0266022.s007.zip › originalgels/E2F-cDNA.jpg]

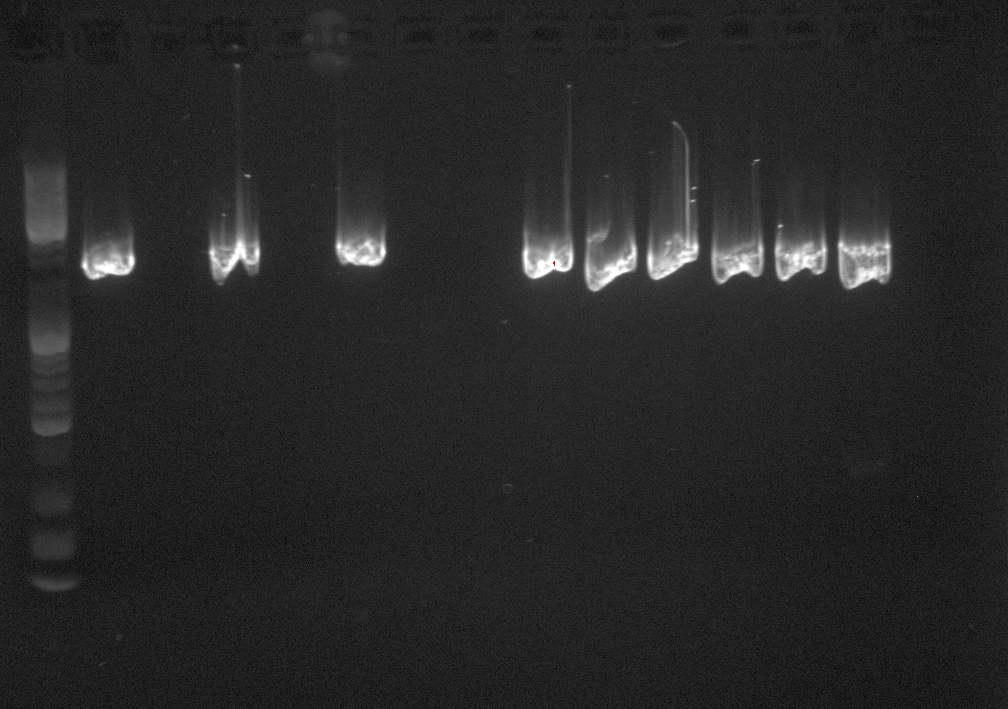

Supplement: S1 Appendix — (ZIP) [file pone.0266022.s007.zip › originalgels/E2F-gDNA.jpg]

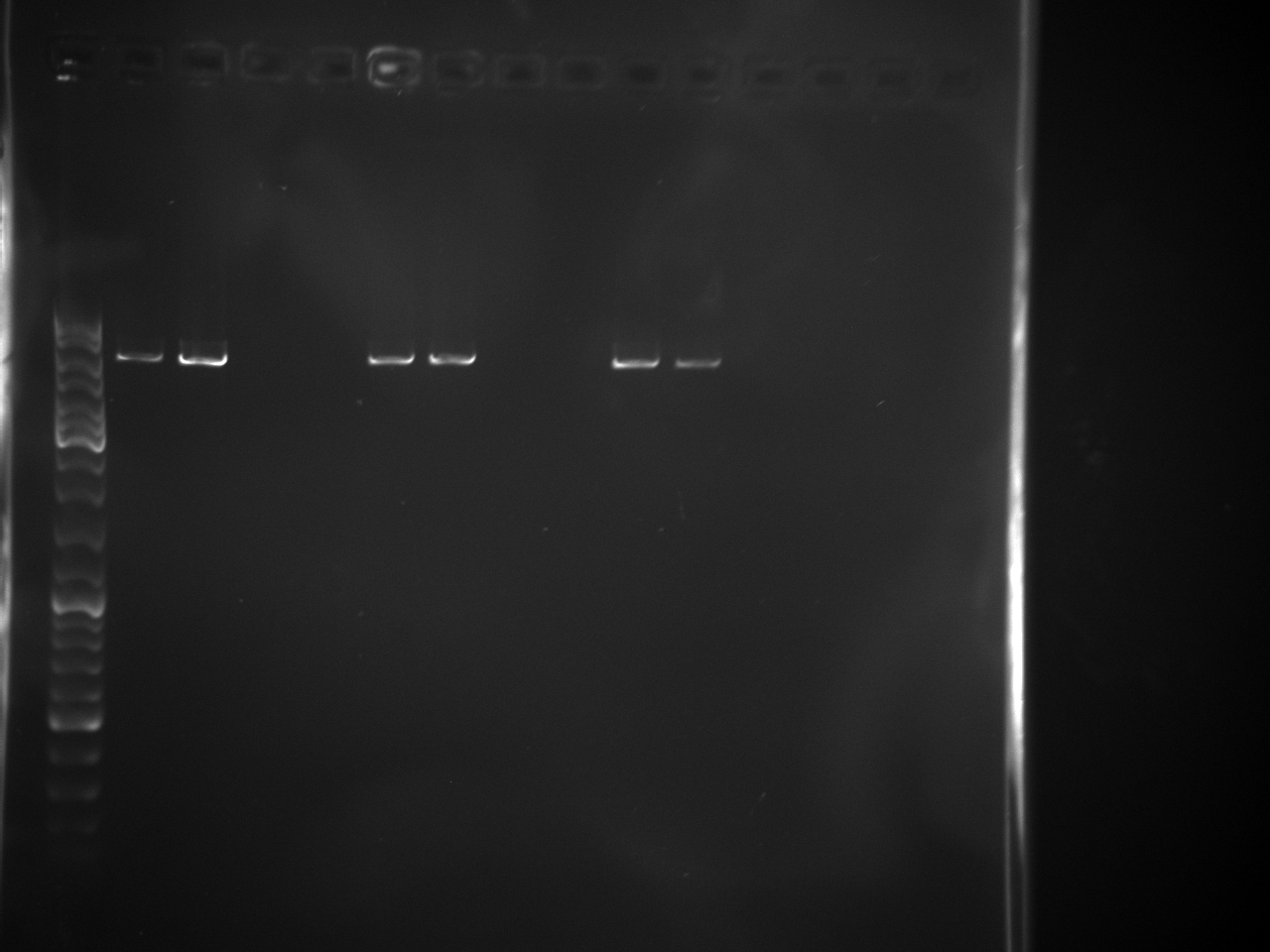

Supplement: S1 Appendix — (ZIP) [file pone.0266022.s007.zip › originalgels/ksr2-femspec.jpg]

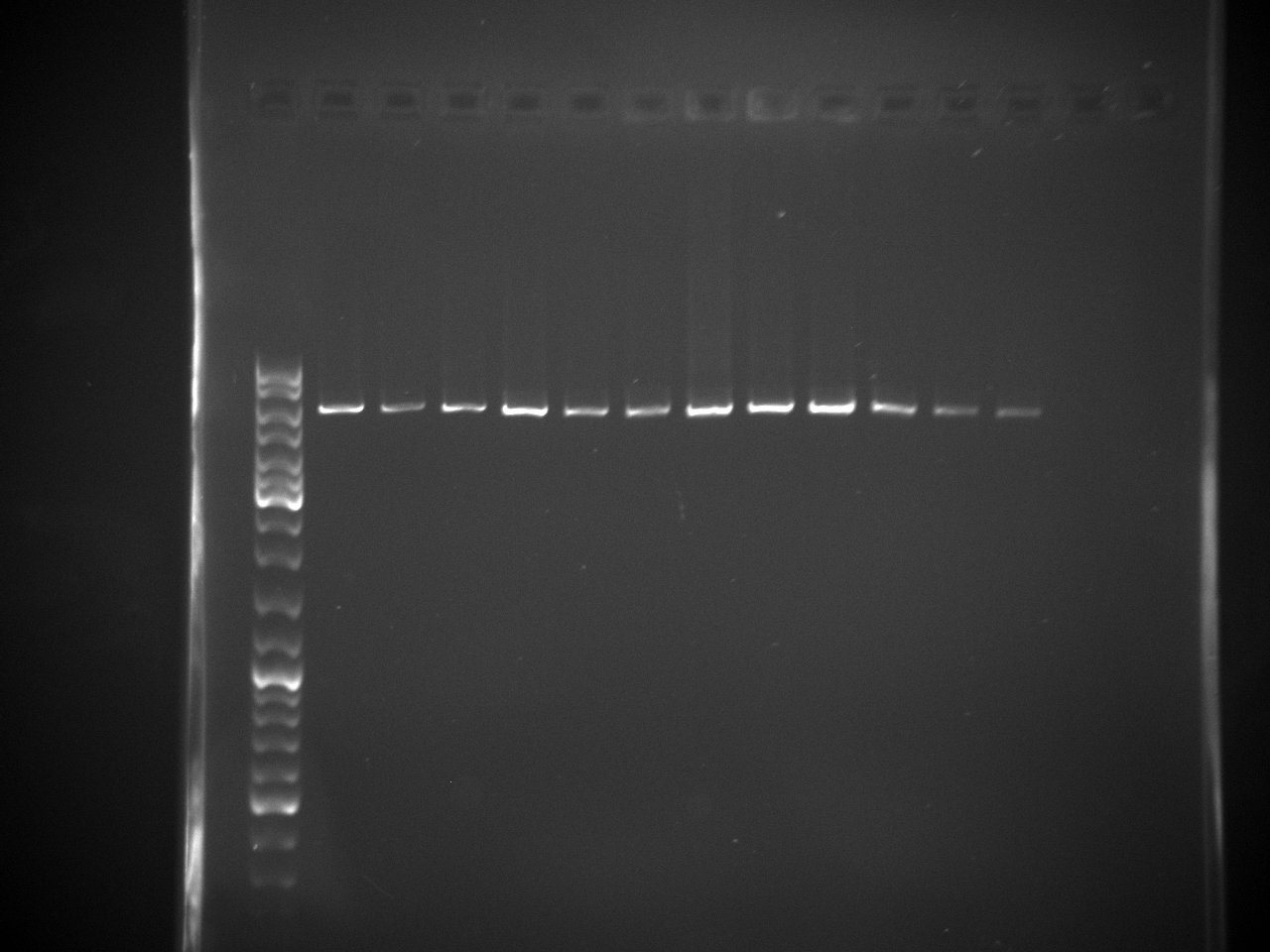

Supplement: S1 Appendix — (ZIP) [file pone.0266022.s007.zip › originalgels/ksr2-unisex.jpg]

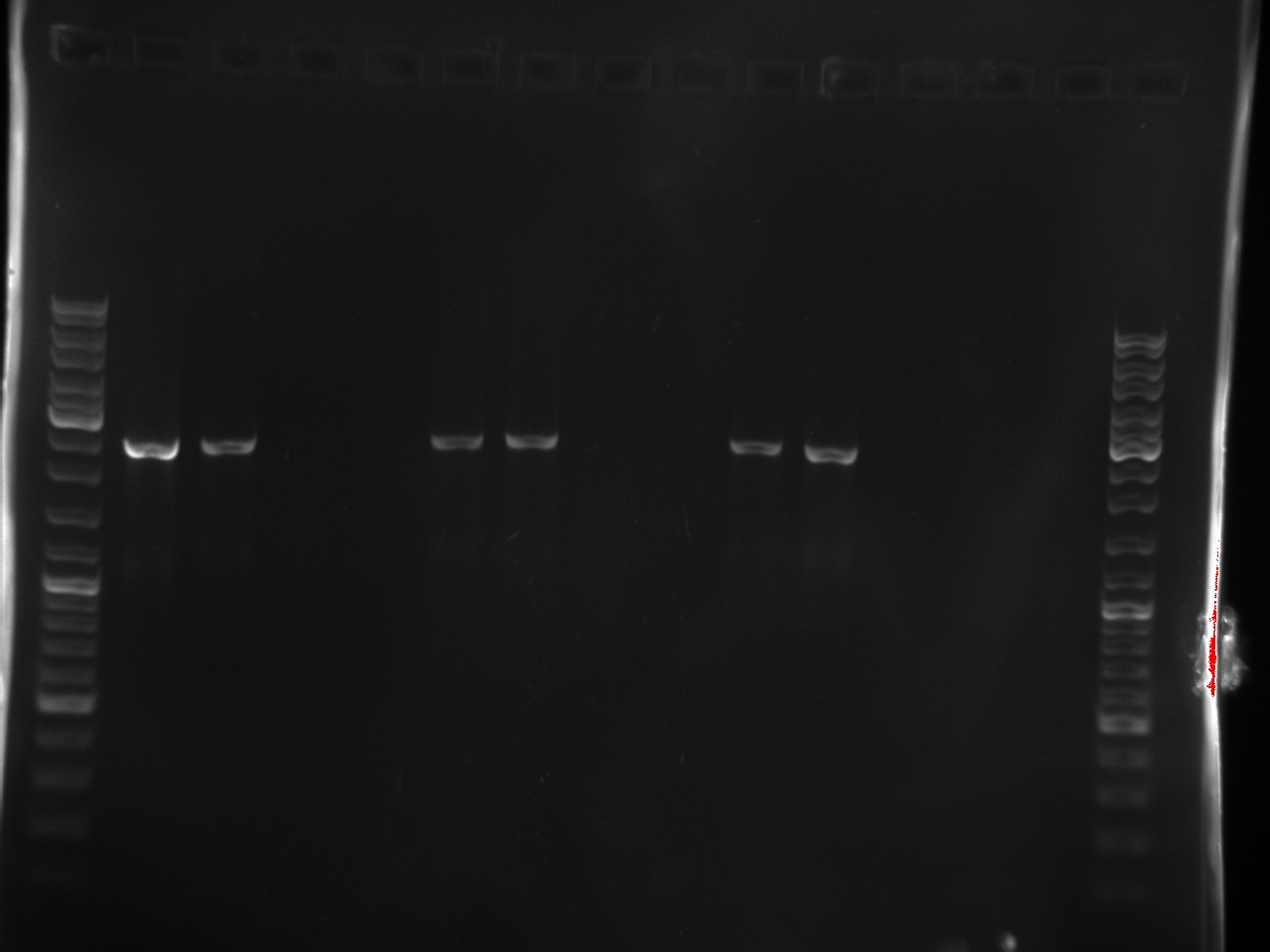

Supplement: S1 Appendix — (ZIP) [file pone.0266022.s007.zip › originalgels/prohibitin-femspec.jpg]

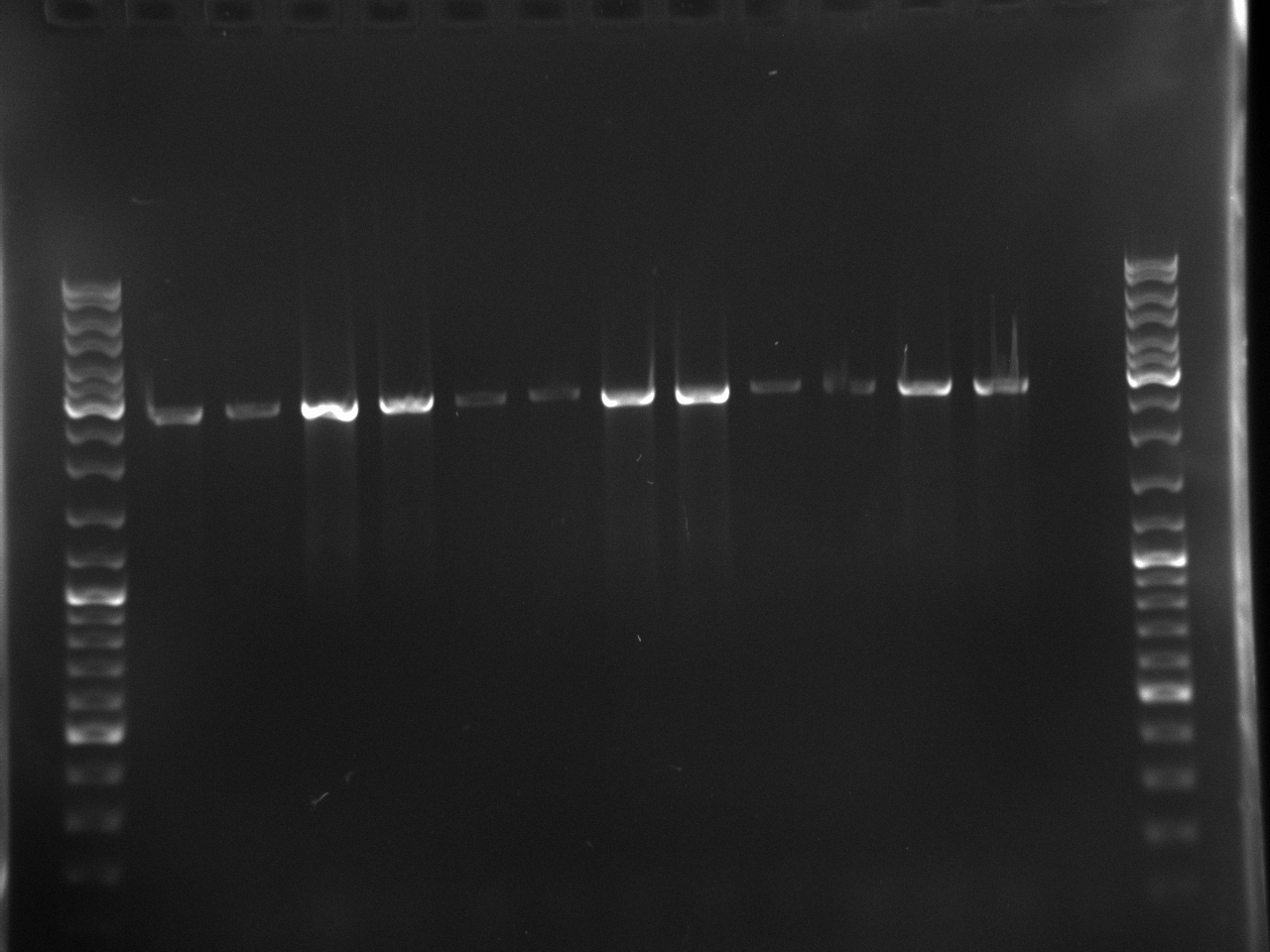

Supplement: S1 Appendix — (ZIP) [file pone.0266022.s007.zip › originalgels/prohibitin-unisex.jpg]
